# Medication Lubricants for Oral Delivery of Drugs: Oral Processing Reduces Thickness, Changes Characteristics, and Improves Dissolution Profile

**DOI:** 10.3390/pharmaceutics16030417

**Published:** 2024-03-18

**Authors:** Marwa A. Malouh, Julie A. Y. Cichero, Yu Sun, Esther T. L. Lau, Lisa M. Nissen, Kathryn J. Steadman

**Affiliations:** 1 School of Pharmacy, The University of Queensland, Brisbane, QLD 4072, Australia; m.abumalouh@uq.edu.au (M.A.M.); j.cichero@uq.edu.au (J.A.Y.C.); yu.sun3@uq.net.au (Y.S.); e.lau@uq.edu.au (E.T.L.L.); l.nissen@uq.edu.au (L.M.N.); 2 Centre for Business and Economics of Health, The University of Queensland, Brisbane, QLD 4072, Australia

**Keywords:** chromophore, polysaccharide, carrageenan, xanthan gum, cellulose gum, rheology, yield stress

## Abstract

Swallowing oral solid dosage forms is challenging for those who have medication swallowing difficulties, including patients with dysphagia. One option is to mix the drug (whole or crushed) with a thick vehicle (medication lubricant). Previous in vitro studies consistently suggest that thick vehicles could impact the dissolution of solid dosage forms, potentially influencing their therapeutic effectiveness, but do not account for changes that happen during oral processing and swallowing. This study aims to investigate the potential impact of medication lubricants on drug release and examine the effect of oral processing. In vitro dissolution of whole and crushed paracetamol tablets mixed with five commercially available medication lubricants (two IDDSI level 2, two IDDSI level 3, and one IDDSI level 4) were tested with and without oral processing; a medication lubricant with/without paracetamol was placed in the mouth (five healthy volunteers), prepared for swallowing, but then expectorated and assessed for physical characteristics and drug release. Medication lubricants, both alone and mixed with crushed paracetamol tablets, showed a significant decrease in viscosity after oral processing. Without oral processing, IDDSI level 3 and 4 lubricants significantly delayed the dissolution of paracetamol tablets. After oral processing, particularly with crushed tablets, there was a substantial increase in the dissolution rate. These findings suggest that dissolution testing overestimates the impact of medication lubricants on drug dissolution. Therefore, using in vitro dissolution tests to predict the dissolution rate of medications mixed with thick vehicles is discouraged. It is essential to consider ways to incorporate the effects of the oral environment and oral processing on thick vehicles used for oral medication administration.

## 1. Introduction

Oral solid dosage forms (i.e., tablets, capsules) are generally designed to be swallowed whole, but many people find this difficult to accomplish. Some people experience a swallowing impairment (dysphagia) as a result of age-related changes in swallowing physiology comorbidities such as stroke, cardiac surgeries, head injury, and polypharmacy [1,2,3]. Food is blended and drinks thickened to produce a consistency that the mouth can safely maneuver into the esophagus rather than the airway [4,5,6], so tablets and capsules can also be unsafe if given whole [7,8]. Individuals with a healthy swallowing function can also struggle to swallow solid oral medications whole [9]. Previous bad experiences with swallowing pills, as well as the physical characteristics of tablets and capsules, i.e., shape, size, and taste, can be factors that lead to this aversion [10].

Using an alternative route of administration or alternative oral formulation, such as liquids or chewable tablets, can be an option [11]. However, the most common way to deal with a whole tablet or capsule that cannot be swallowed whole is to modify it, i.e., crush tablets or open capsules and mix them with food substances or commercial dysphagia-oriented products [9,12]. Around 25% to 33% of medication administration for community-dwelling older patients involved modification of the dosage form to facilitate swallowing [13].

Medication lubricants are a relatively recent product group that has been commercialized. These products were originally designed for use by anyone who struggles to swallow tablets whole using water and who may find that a thicker, lubricating substance better masks the tablet and makes it easier to swallow whole [14]. They are being used to help delivery of both whole and crushed medications. These products are composed of plant gums that form polysaccharide networks. However, it has been demonstrated that mixing crushed medications with dysphagia-oriented thickeners, which are also composed of natural polysaccharide gums that form polymer chain networks, has the potential for a significant delay in disintegration and dissolution [15,16]. For instance, mixing crushed tablets of atenolol with xanthan gum-based thickener showed restriction in drug release in vitro testing, exhibited only 50% release by 30 min, and took 3 h to reach 85% [16]. There is a concern that medication lubricants might impede drug release and therefore impair drug bioavailability.

The first aim of this study is to evaluate the effect of medication lubricants on the drug dissolution of crushed and whole tablets. The in vitro dissolution test is routinely used to provide information about drug-release characteristics and to predict in vivo absorption profiles [17,18]. Additionally, this method has been used in previous research to assess the influence of mixing crushed medications into dysphagia-oriented thickening agents on drug absorption [15,16,19,20]. However, in a previous study, the relationship between in vitro drug release and in vivo drug absorption was not directly aligned. The in vivo absorption of crushed paracetamol tablets mixed with xanthan gum-based dysphagia-oriented thickeners was substantially faster than what was predicted from in vitro dissolution tests [21]. It has been suggested that this is associated with changes that occur to the bolus structure during deglutition that change the form of the bolus by the time it reaches the stomach, which, in turn, might increase drug release [11,22].

Deglutition is a sequence of voluntary and involuntary reflexes and movements managed by the central nervous system [23]. The oral phase involves various oral operations, including first bite, molding, manipulation, saliva incorporation, mastication, equilibration to the oral cavity temperature, bolus formation, and bolus transportation to the back of the oral cavity to be swallowed. These result in mechanical and physical changes to the bolus structure. The main enzyme in saliva, α-amylase, hydrolyses starch and breaks it down into a simple carbohydrate [24]. Therefore, the mechanical structures of starch-based food and drinks (e.g., custard) are broken down and their viscosity is reduced to less than half within the first 10 s in contact with human saliva [25]. Similarly, the viscosity of starch-based thickeners is affected by deglutition, which consequently threatens swallowing safety for patients with dysphagia [26,27].

By analyzing the physical properties of the bolus during oral processing, a significant decrease in hardness and an increase in springiness, adhesiveness, and cohesiveness of cereal bolus after oral manipulation has been observed [28]. Shearing and elongation of the bolus during transportation through the deglutition process are anticipated to be factors leading to deformation [29,30]. The bolus becomes extremely stretched and deformed while it is transported from the oral cavity to the stomach [31].

Therefore, the second aim of this study is to investigate the effect of oral processing on the physical characteristics (viscosity and texture features) of the medication lubricants and whether it affects the dissolution of whole and crushed tablets mixed with medication lubricants. In this study, the in vivo approach is used, which involves the medication lubricant with/without a paracetamol tablet being placed in the mouth of healthy individuals and prepared for swallowing, and just prior to being swallowed the bolus is collected and characterized in terms of viscosity, texture features, and drug dissolution profile.

## 2. Materials and Methods

### 2.1. Materials

Immediate-release 500 mg paracetamol tablets (Panamax, Sanofi Aventis Australia, Macquarie Park, NSW, Australia) were used. Paracetamol is classified in the Biopharmaceutical Classification System (BCS) as class III but possesses some attributes of BCS class I [32]. Paracetamol was used in this study because it has a pKa value of 9.4, which ensures that at the range of pHs encountered in physiological conditions, its solubility is pH-independent [33]. Therefore, the very acidic stomach conditions (pH = 1.2) we used in vitro and the slightly acidic oral environment would have minimal impact on paracetamol solubility. Also, paracetamol is the most frequently modified tablet in Australian hospitals and is most frequently reported as difficult to swallow [34,35]. Moreover, paracetamol has a chromophore within the chemical structure that enables detection by UV spectroscopy.

Five commercial medication swallowing lubricants were tested: MediSpend, Severo Swallowing Gel, Gloup Low Sugar, Gloup Original, and Gloup Forte (Table 1). All are ready-to-use products that are placed onto a spoon, and the pill is placed into it before swallowing [14]. All products were tested at room temperature (approximately 24 °C).

### 2.2. Experimental Design

The effect of medication lubricants on drug dissolution (aim 1) was tested by comparing paracetamol dissolution for whole or crushed tablets with or without one of the five medication lubricants. The influence of oral processing on the lubricant’s physical characteristics and the rate of drug dissolution (aim 2) was investigated by including oral processing in a human mouth prior to testing in the dissolution apparatus, rheometer, and texture analyzer. All measurements were applied to five replicate samples of each medication lubricant.

### 2.3. Sample Preparation

For each medication swallowing lubricant, 10 g was weighed into a 30 mL plastic cup. This was derived by weighing the manufacturer’s recommended quantity and found to be a reasonable approximation for experimental purposes.

For tests using whole tablets, one whole paracetamol tablet was placed into the center of the lubricant within the cup. For tests using crushed tablets, one tablet was crushed using a mortar and pestle before transferring into the plastic cup and stirred with a plastic spatula for 30 s to incorporate it into the medication swallowing lubricant.

Loss of crushed paracetamol weight during transferring samples containing crushed tablets was measured; the pestle and mortar, weighing boats, and plastic cups that were used for crushing, mixing, and transfer processes were rinsed; the rinse was filtered and diluted; the absorbance was measured; and then the amount of paracetamol was calculated. The yield of the sample preparation was calculated without accounting for the drug lost during the preparation process.

### 2.4. Oral Processing Effect

Five healthy participants were recruited in this experiment, two males and three females, with ages ranging from 24–49 years. All participants provided written consent to participate in the experiment and reported no history of allergies to paracetamol. Participants who were smokers, pregnant, or breastfeeding were excluded from this study. This study was approved by the University of Queensland Human Research Ethics Committees (approval number: 2019000338). Participants were asked not to consume any form of paracetamol for 4 h before the experiment and refrain from eating or drinking (except water) 60 min before the study. The five participants attended this experiment on five occasions. On each visit, the participants were asked to test samples containing one of the five medication lubricants.

The participant took each sample (samples prepared as described in Section 2.3) into the mouth and prepared it for swallowing. The participants were instructed to spend no less than 5 s and no more than 10 s for sample manipulation in the mouth. Previously, it has been reported that healthy individuals spend around 5 s manipulating a starchy liquid in the mouth and around 10 s for semi-solid starchy foods [36]. When the participants felt that the bolus was ready to be swallowed, they spat it out into a plastic cup. The expectorated samples were immediately tested in a dissolution apparatus, rheometer, and texture analyzer. The participants extensively rinsed their mouths with water between samples.

### 2.5. Drug release and Dissolution

The samples were tested using a 708-DS dissolution apparatus (USP rotating paddle (apparatus 2)) and an 850-DS Dissolution Sampling Station connected to workstation software (Agilent, Mulgrave, Victoria, Australia). The dissolution medium was 900 mL of simulated gastric fluid (SGF) at pH 1.2 without enzymes [37], under sink conditions, mixed with a paddle rotation speed of 50 rpm at a medium temperature resembling a human body temperature of 37 °C. Samples of 1 mL were collected at 5, 10, 15, 20, 25, 30, 45, 60, 90, 120, 150, and 180 min through a 10 µm filter and transferred to glass tubes by the dissolution apparatus. Then, 100 µL of each sample was transferred to a plastic tube and diluted with 5 mL of SGF. The absorbance of each sample was measured at 245 nm using a Cary 60 spectrophotometer (Agilent, Mulgrave, VIC, Australia). Fresh SGF (1 mL) was replaced by the apparatus at every sampling point [38].

To consider the background absorbance associated with medication-swallowing lubricants, the calculated concentration of medication-swallowing lubricants without the drug (control) was subtracted from the concentration of the samples containing paracetamol. The cumulative percentage of paracetamol that dissolved in the samples was plotted against time. Measurements were carried out for five replicate samples of each medication lubricant.

Standard solutions were prepared in the range of 2–12 μg/mL. This was prepared from a stock solution; approximately 50 mg of paracetamol (acetaminophen, Sigma Aldrich A7080-100G, lot SLCB2770, Sigma, St. Louis, MO, USA) was weighed and dissolved in 100 mL of SGF (pH 1.2) to obtain a concentration of 500 μg/mL. Aliquots of filtered stock in the range of 0.4 to 2.4 mL were diluted with SGF to prepare standard solutions with concentrations of 2, 4, 6, 8, 10, and 12 μg/mL. To prepare the sample solutions for method validation, ten paracetamol tablets were precisely weighed and crushed. An amount of powder equivalent to 500 mg of paracetamol was taken from the crushed tablets to prepare the stock and dilutions, using the same method as for the standard solutions.

The method was validated for specificity, linearity, sensitivity (detection limit and quantitation), precision (repeatability and intermediate precision), and accuracy as stated by the International Conference on Harmonisation of Technical Requirements for Registration of Pharmaceuticals for Human Use (ICH) [39]. To evaluate the specificity, the UV scan of a blank solution (SGF mixed with one of the medication lubricants at a time, including MediSpend, Severo, Gloup Low Sugar, Gloup Original, and Gloup Forte) was compared with that of standard and sample solutions containing 11 μg/mL of paracetamol across a range of 200 to 500 nm. This was done to ensure that there were no interfering peaks from the blank solutions or tablet excipients and to determine the wavelength of maximum absorption for paracetamol, which was found to be 245 nm.

A six-point calibration curve was prepared with standard solutions in triplicate. A linear relationship was obtained within the concentration range of 2–12 μg/mL at a wavelength of 245 nm. The validity of the calibration model was supported by the correlation coefficient, y-intercept, slope of the regression line, and residual sum of squares (*y* = 0.0654*x*, *r*^2^ = 0.999). To determine the sensitivity of the method, the limit of detection (LOD) and the limit of quantification (LOQ) were calculated. The LOD was found to be 2.1 μg/mL and the LOQ was 6.25 μg/mL. These values were calculated using the residual standard deviation of the regression line (σ) and slope (S) of the calibration curve, with the formula for LOD being 3.3σ/S and LOQ being 10σ/S.

Precision was assessed in terms of repeatability (intra-day) and intermediate precision (inter-day) by determining the standard deviation (SD) and relative standard deviation (% RSD) around the mean of six repeated measurements taken on the same day (for repeatability) or two consecutive days (for intermediate precision) for a sample solution at a concentration of 11 μg/mL. Accuracy was determined by performing recovery tests in triplicate at 80%, 100%, and 120% of the test concentration. To conduct these tests, amounts of crushed paracetamol tablets equivalent to 400 mg, 500 mg, and 600 mg of paracetamol were transferred into a 500 mL volumetric flask, diluted, and measured. The SD for intra-day precision was 0.38 and 0.24–0.45 for inter-day precision. The % RSD for intra-day precision was 3.57%, while the range of inter-day precision was 2.21–4.41%. The recovery range was found to be 90.4–96.2%.

Two-way ANOVA followed by a Dunnett’s multiple comparisons test was used to determine the significance of differences in the quantity of drug dissolved at 30 min for tablets in swallowing lubricant compared to the control tablets, using GraphPad Prism version 8.00 (San Diego, CA, USA). Differences were considered significant at *p* < 0.05. Results are given as mean and standard error for five replicates.

### 2.6. Viscosity Measurements

Viscosity measurements of the samples were performed using a stress-controlled rheometer HR-3 (TA instruments C/O Waters Australia, Sydney, NSW, Australia) with parallel plates. All samples were tested at room temperature (24 °C). All measurements were carried out through the peak hold test at 24 °C with a shear rate of 50 s^−1^ over a period of 60 s. This shear rate is commonly used to evaluate liquid foods as an indicator of the shear rates of swallowing, and using this value facilitates comparison between studies [40,41]. The gap ranged from 200 to 500 µm for samples without paracetamol and 600 to 900 µm for samples containing crushed paracetamol, depending on the gap suitability for each lubricant. Medication lubricant samples without oral processing were tested as a control.

One-way ANOVA followed by a Bonferroni post hoc comparisons test was performed to study the effect of oral processing and adding crushed tablets on the viscosity of each medication lubricant. Analyses were performed using GraphPad Prism version 8.00 (San Diego, CA, USA), with differences considered significant at *p* < 0.05. Results are given as mean and standard error for five replicates.

### 2.7. Texture Features Measurements

A Brookfield Texture Analyser CT3 (Ametek Brookfield, Middleboro, MA, USA), equipped with a 4500 g load cell and texture analysis software program, was used to determine the texture properties of the medication lubricants (adhesiveness gumminess, hardness, springiness, and cohesiveness). Measurements were made at room temperature (24 °C). Texture profile analysis (TPA) [42] was performed using a clear cylinder probe of 12.7 mm diameter and 35 mm length (TA10). A 27 g of the medication lubricants was filled in a standard beaker (40 mL). The sample was compressed at pretest, test, and return speeds of 2, 1, and 1 mm s^−1^ to a distance of 20 mm. A force of 0.1 g was used as a trigger value. Medication lubricant samples without oral processing were tested as a control.

For each medication lubricant, differences in texture measurements with and without oral processing, and with and without crushed drug, were analyzed by one-way ANOVA followed by a Bonferroni multiple comparisons test. Analyses were performed using GraphPad Prism version 8.00 (San Diego, CA, USA). Results are given as mean and standard error for five replicates. Differences were considered significant at *p* < 0.05.

## 3. Results

### 3.1. Dissolution Experiment

In this study, the loss of crushed paracetamol weight was between 1% and 5% (Table 2). This indicates that the maximum percentage of dissolution that could have been measured from crushed paracetamol tablets was 95% to 99%; after 30 min in the dissolution test, crushed tablets measured 95.8% and so can be presumed to have fully dissolved. For whole tablets, for which we presume 100% is the potential maximum dissolution, 91 ± 3.1% had dissolved at 30 min (Table 3).

**Figure 1:**
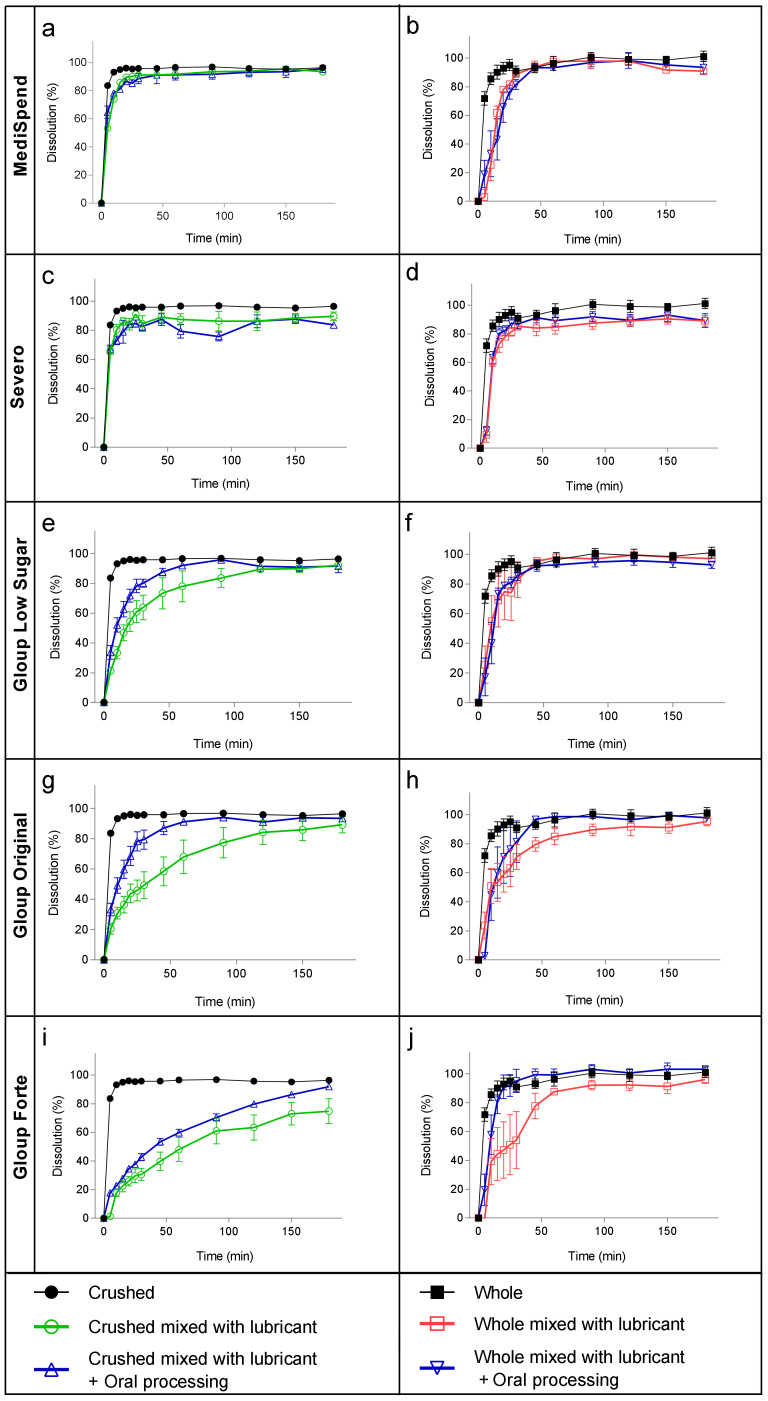
Dissolution of paracetamol mixed with MediSpend (**a**,**b**), Severo (**c**,**d**), Gloup Low Sugar (**e**,**f**), Gloup Original (**g**,**h**), and Gloup Forte (**i**,**j**), with and without oral processing, in simulated gastric fluids using crushed tablets (**a**,**c**,**e**,**g**,**i**) and whole tablets (**b**,**d**,**f**,**h**,**j**). The bars indicate mean ± se for five replicates.

The samples of crushed tablets mixed with the thinnest medication lubricants, MediSpend and Severo, exhibited very rapid dissolution in SGF, with 86% of the drug released in the first 15 min (Figure 1a,c). Consequently, at 30 min, the dissolution of crushed tablets from these lubricants was not significantly different from the dissolution of control crushed tablets (Table 3). In contrast, the release of crushed paracetamol from the moderately thick lubricants (IDDSI level 3) Gloup Original and Gloup Low Sugar was delayed, with only 49% and 64%, respectively, released at 30 min, which was significantly different (*p* < 0.001) to the control crushed tablet (Table 3). Gloup Forte (extremely thick, IDDSI level 4) restricted the drug release the most, reaching only 75% dissolution after 180 min (Figure 1i). Therefore, there was a statistically significant restriction in drug release at 30 min (*p* < 0.001) (Table 3). It was observed that the thinnest medication lubricant samples (MediSpend and Severo) broke into small pieces as soon as they were added to the vessels of the dissolution apparatus, whereas the samples of moderately or extremely thick (IDDSI level 3 and 4) medication lubricants remained as a single mass at the bottom of the vessel for the whole experiment. FDA guidance indicates that the amount of drug release from immediate-release tablets in the dissolution test should not be less than 85% within 30 min [43], but this was not achieved by crushed paracetamol mixed with IDDSI level 3 or 4 medication lubricants (Figure 1g,e).

Whole tablets also showed a fast dissolution profile when delivered with the thinnest medication lubricants (MediSpend and Severo), reaching more than 85% within 30 min. The dissolution profile of whole tablets mixed with moderately thick (IDDSI level 3) medication lubricants reached 85% when delivered with Gloup Low Sugar within 30 min, and although dissolution from Gloup Original was only 71% at 30 min, this was not significantly different to the control whole tablet according to the ANOVA analysis (Table 3). Gloup Forte (IDDSI level 4) was the only lubricant that caused a statistically significant delay in drug release for whole tablets compared with the control (*p* < 0.05; Table 3), though this was associated with considerable variation between replicates. The variation between replicates in this and other measurements with whole tablets is likely to be associated with the observation that for some samples, the whole tablets dropped out of the medication lubricant lump immediately after adding them to the dissolution vessel, while others remained trapped in the lubricant for a period of time, which delayed tablet disintegration. Oral processing had a large effect on the dissolution of paracetamol from both moderately thick (IDDSI level 3) and extremely thick (IDDSI level 4) medication lubricants, with considerably higher quantities of paracetamol being released by 30 min for both whole and crushed tablets (Table 3). Following oral processing, only the release of paracetamol from crushed tablets in Gloup Forte (IDDSI level 4) was significantly lower than the control at 30 min (*p* < 0.05; Table 3). Indeed, it was observed that after oral processing, the samples mixed with moderately and extremely thick medication lubricants broke into smaller pieces when added to the dissolution vessels. It appears that oral processing of the samples facilitated the dropping out of whole tablets of the medication lubricants lump in the dissolution vessels, resulting in a decrease in the variation between replicates of drug release of whole tablets.

### 3.2. Viscosity Measurements

**Figure 2:**
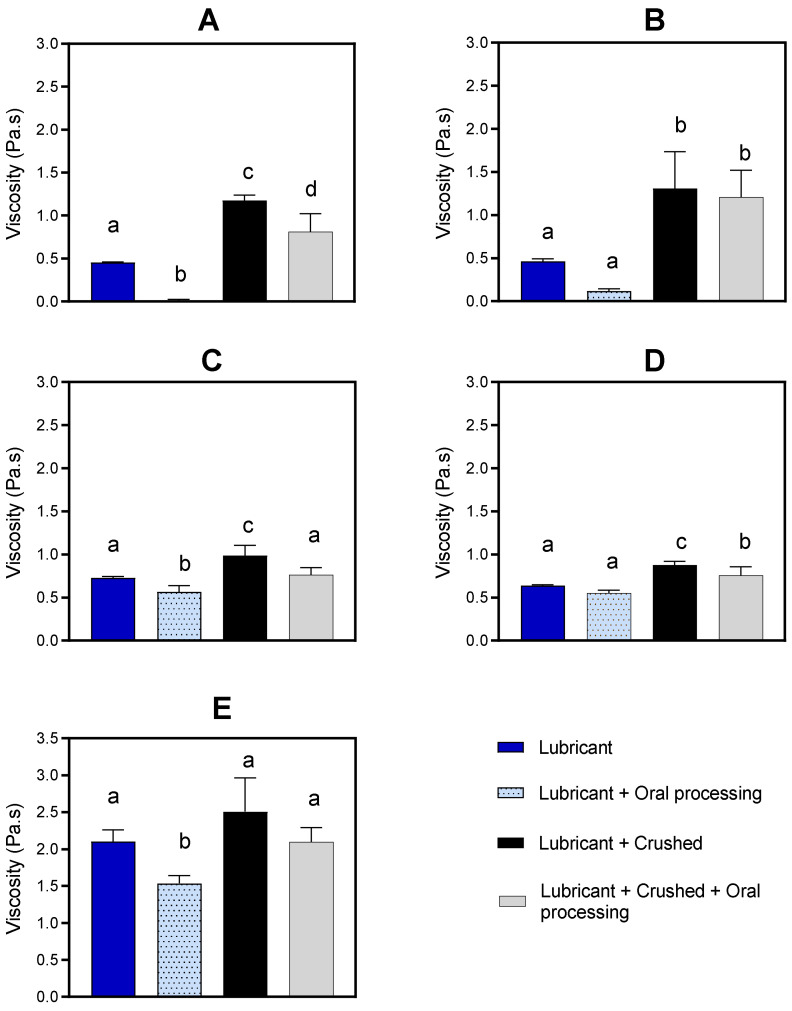
Viscosity (Pa·s) of Medispend (**A**), Severo (**B**), Gloup Low Sugar (**C**), Gloup Original (**D**), and Gloup Forte (**E**) with and without crushed tablets and/or oral processing were measured at 50 s^−1^. For each medication lubricant analyzed separately using ANOVA with Bonferroni post hoc comparisons, bars with unlike superscript letters above are significantly different (*p* < 0.05). The data are mean ± se for five replicates.

The viscosity of the medication lubricants increased when crushed paracetamol tablets were incorporated into them (Figure 2). The increase ranged between 0.24 and 0.92 Pa·s and was most obvious for the thinnest medication lubricants (MediSpend and Severo), which increased to viscosity values similar to those of moderately thick products (IDDSI level 3: Gloup Low Sugar, Gloup Original).

The viscosity of the medication lubricants generally decreases as a result of oral processing. For medication lubricants alone, without crushed tablets incorporated, there was a reduction of between 0.09 and 0.56 Pa·s (Figure 2). For medication lubricants with crushed paracetamol tablets incorporated, there was a reduction in viscosity measurements of between 0.12 and 0.41 Pa·s.

### 3.3. Texture Features Measurements

The effects of oral processing and the addition of crushed tablets on the texture features of the medication lubricants are summarized in Table 4. Overall, incorporating crushed tablets into the lubricants without oral processing resulted in a decrease in hardness, gumminess, and springiness. The reduction of hardness was statistically significant for MediSpend, Severo, and Gloup Forte, while the reduction of gumminess was statistically significant for MediSpend, Gloup Original, and Gloup Forte, and the reduction of springiness was statistically significant for Gloup Original and Gloup Forte, with other lubricants exhibiting a similar trend. However, the effects of oral processing negated the effect of adding crushed tablets, as the measurements reverted to values similar to the control after oral processing. In general, there was a decreasing trend in the springiness and gumminess of medication lubricants without crushed tablets after oral processing. However, no significant changes were observed in adhesiveness or cohesiveness after adding crushed tablets or undergoing oral processing.

## 4. Discussion

This study examined the changes in viscosity and texture features of medication lubricants following oral processing. Results showed a decrease in the viscosity of medication lubricants alone (ranging from 0.09 to 0.56 Pa·s) and for medication lubricants mixed with crushed paracetamol tablets (ranging from 0.12 to 0.41 Pa·s) after oral processing. Additionally, a decreasing trend in springiness and gumminess was observed in medication lubricants alone after oral processing. These changes can be attributed to the complex manipulations that substances undergo during oral processing, including dilution, mechanical and enzymatic breakdown, and clustering or softening of substance particles [44,45]. Oral processing also involves equilibration to oral cavity temperature, the mechanical effects of tongue and teeth, and conversion and formation processes that convert the bolus into a rheologically suitable state for swallowing [44,45,46].

We suggested that the changes that occur to the bolus during oral processing play a crucial role in facilitating drug release for solid oral medications administered with thick vehicles such as food vehicles, thickening agents, and medication lubricants. In addition to oral processing, the swallowing process, including bolus deformation potentially occurring through shearing in the pharynx and esophagus and during gastric mixing and emptying, can facilitate drug release [47,48,49]. Therefore, the reliability of using dissolution testing alone to predict the effect of thick vehicles, including medication lubricants, on drug disintegration and dissolution without mimicking the effect of the oral processing on the bolus should be questioned. And so, in this study, we investigated the impact of oral processing on the dissolution profile of paracetamol tablets mixed with medication lubricants using the dissolution testing method.

The dissolution testing results, without prior oral processing, showed that the moderately thick medication lubricants (Gloup Original and Gloup Low Sugar) and the extremely thick medication lubricant (Gloup Forte) significantly delayed the release of paracetamol tablets. Gloup Forte, which has the highest viscosity (2.1 Pa·s) (Figure 2) and is classified as IDDSI level 4, which is the thickest consistency of fluids in the IDDSI international guideline, showed the highest restriction of paracetamol dissolution. Drug release after 30 min was only 31% for crushed tablets and 54% for whole tablets. For Gloup Low Sugar and Original (0.73 Pa·s and 0.64 Pa·s, respectively), which are classified as IDDSI level 3, dissolution was slowed to a lesser extent, as paracetamol released after 30 min were 49–64% for crushed tablets and 71–86% for whole tablets. These results agree with the previous findings, which showed that mixing crushed medications with thickened fluids or thick food (i.e., jam, honey, and yoghurt) that are commonly used as drug vehicles for dysphagic patients, had a significant influence on in vitro drug dissolution [15,16]. In previous work, we found that vehicles with higher apparent yield stress and greater solid-like rheological behavior (higher viscoelasticity) were mostly associated with the most significant restriction of drug release [15,16]. For the products with a higher yield stress, higher shearing stress is required to break down the structure of lubricants in the in vitro dissolution test. This agrees with our results as Gloup Forte, which has the greatest yield stress (38.13 Pa), caused the greatest restriction in paracetamol release, while MediSpend, which has the lowest yield stress (2.47 Pa), was associated with the least restriction.

With oral processing prior to dissolution testing, there was a significant increase in the dissolution rate of paracetamol tablets when mixed with moderately and extremely thick medication lubricants. This increase was more obvious with the crushed tablets than the whole tablets. The release of crushed paracetamol mixed with Gloup Low Sugar reached 85% within 45 min instead of 90 min and within 45 min instead of 120 min for Gloup Original. For Gloup Forte after oral processing, it reached 85% after 150 min, while without oral processing it reached only 75% after 180 min. This improvement in drug release is predicted to be due to the changes that occurred to the samples during oral processing. It was observed that after oral processing, the samples mixed with moderately and extremely thick medication lubricants broke into smaller pieces when added to the dissolution vessels. Also, it is most likely that the decrease in the viscosity of the medication lubricants after oral processing played a role in improving the paracetamol dissolution profile. These results suggest that the impact of moderately and extremely thick medication lubricants on drug dissolution rate may be overestimated by dissolution testing. This is because in vitro dissolution tests are designed for assessing whole tablets that remain intact upon arrival in the stomach, and are not suitable for predicting drug release when tablets are mixed with thick vehicles or crushed, due to the lack of consideration for the changes that occur during the oral and swallowing process. Therefore, relying on in vitro dissolution testing alone to predict the release of medications mixed with thick vehicles, including medication lubricants or thickened fluids is not recommended.

Regardless of whether oral processing was performed prior to dissolution testing or not, Severo and MediSpend, the thinnest medication lubricants, showed a relatively low viscosity (0.467 Pa·s and 0.453 Pa·s) and had no significant impact on paracetamol dissolution profile. Mixing crushed medication (solids) into liquids with a continuous flow property creates a mixed consistency. When liquids are chewed or when people consume mixed consistencies of solids plus liquids, a portion of the bolus reaches the pharynx before the onset of swallowing. For people with dysphagia, this increases the risk of aspiration of material that has come to rest in the pharynx before the swallow and before the airway is closed during swallowing [50]. Although thick fluids that flow continuously do not appear to affect drug release, when mixed with crushed medications (solids), the safety of these products for patients with dysphagia is a concern.

Biopolymers (i.e., carrageenan, xanthan gum, cellulose gums) are usually used as thickening agents for dysphagia-oriented products, primarily due to their rheological properties that allow the formation of viscous fluids [51]. These products’ magnitude of solution viscosity and thickness level mainly depends on the polymer concentration [52,53]. Therefore, extremely and moderately thick medication lubricants (Gloup Forte, Gloup Low Sugar, Gloup Original), which showed a significant restriction in drug release in in vitro dissolution test, are most likely to contain higher concentrations of biopolymer than thinner products (Severo, MediSpend). Therefore, it is highly expected that Severo and MediSpend would show higher restriction in drug dissolution rate if they had a higher biopolymer concentration.

Medication lubricants were originally designed to help people swallow tablets/capsules as a whole and therefore reduce the need to crush tablets. Whole tablets exhibited a faster drug release rate than crushed tablets. When whole tablets were mixed with Severo, MediSpend, and the Gloup original medication lubricants, no significant delay in drug release was observed compared to the control. These findings suggest that administering whole tablets/capsules with medication lubricants, may not significantly impede drug release and can potentially serve as a potential option for people with only a medication swallowing difficulty. Only the extremely thick lubricant (Gloup Forte) caused a statistically significant delay in drug release from whole tablets. Despite this delay, the dissolution profile of whole tablets mixed with Gloup Forte was faster than that of crushed tablets (30.6% at 30 min compared to 54% at 30 min, respectively). However, considerable variation in drug release rates was observed between replicates of whole tablets, which can be attributed to the inconsistent dropping out of tablets from the medication lubricant lumps in the dissolution vessels. The observed fragmentation of samples into smaller pieces following oral processing facilitated the dropping out of whole tablets from the medication lubricant lumps in the dissolution vessels, resulting in a decrease in variability between replicates.

Despite being originally designed to aid in swallowing whole tablets, medication lubricants are reportedly being used as a vehicle for administering crushed medication to aged care residents with swallowing difficulties, including dysphagic patients. Incorporating crushed tablets into the medication lubricants resulted in changes in viscosity and some of the texture features of these lubricants. An increase (ranging between 0.24 and 0.92 Pa·s) in the viscosity was measured across all five medication lubricants with and without oral processing. Adding crushed tablets to the thinner medication lubricants, MediSpend and Severo, resulted in a noticeable increase in viscosity, bringing the values of these products closer to those of moderately thick products. This increase in viscosity may not pose a concern for patients with dysphagia; however, a case-by-case assessment from a speech pathologist would be required to determine whether the product was suitable for individuals with dysphagia. Overall, incorporating crushed tablets into the lubricants without oral processing resulted in a decrease in hardness, gumminess, and springiness. Evidence shows that bolus with high gumminess of the fluids tends to stick to the inner part of the mouth and pharynx and therefore increases the risk of aspiration [54,55,56]. Food with high hardness that is unlikely to disintegrate, such as a solid dose medication (tablet/capsule), might be associated with an increase in the risk of aspiration [56,57]. Therefore, opening capsules and crushing uncoated or compressed tablets that pharmacokinetically presented no risk or low risk for crushing and mixing it with a thick vehicle might be a reasonable option to ease medication administration.

MediSpend, one of the thinner medication lubricants, is a modified food starch-based lubricant. It showed the greatest reduction in viscosity (from 0.453 to 0.011 Pa·s) after oral processing. This reduction is unsurprising since it is demonstrated that starch-based products are susceptible to a dramatic decrease in viscosity after contacting human saliva as a result of α-amylase enzyme activities [58,59]. Although gum-based products are not predicted to be broken down by α-amylase enzyme, they have been observed to be susceptible to a reduction in viscosity after contacting human saliva [27,60]; alternatively, a small volume of saliva is added during oral processing and this dilutes the bolus and makes it thinner. This justifies the significant reduction in viscosity measurements of non-starch-based products (Gloup Low Sugar, Gloup Forte) after contact with saliva. In healthy individuals, oral processing for liquids is approximately 1–2 s. For this experiment, oral processing occurred for 5–10 s, which is in the range of individuals with significant dysphagia [61], and allows reasonable infusion and incorporation of saliva into the sample. The significant reduction in viscosity during oral processing could compromise the safety of swallowing since the patient does not receive the prescribed thickness level. Speech pathologists should evaluate oral processing time and observe the administration of crushed medication with a medication lubricant before recommending it for that individual. Further research is required to evaluate the effect of viscosity alteration during oral processing on the safety of swallowing for patients with dysphagia. More research is needed to consider other factors that might cause further improvement in the dissolution profile of drugs when mixed with moderately and extremely thick medication lubricants, including bolus deformation that potentially occurs through the pharynx and esophagus, and during gastric mixing and emptying. As demonstrated by this study, the dissolution test is most likely to overestimate the impact of moderately and extremely thick medication lubricants on drug dissolution rate. Consequently, further research should explore whether these products significantly affect the rate and extent of drug absorption in vivo.

However, in this study, there is a limitation in recruiting healthy participants rather than patients with dysphagia. The disease state and prescribed medications used by patients with dysphagia are usually associated with salivary gland hypofunction and severe deficit in tongue pressure and control [62,63], probably affecting the patient’s ability to mix a bolus with saliva and alter the duration of the oral preparation phase.

## 5. Conclusions

The current study results indicated that substantial changes in dissolution profile and medication lubricant characteristics had been shown after oral processing, suggesting that the dissolution test is most likely to overestimate the impact of moderately and extremely thick medication lubricants on drug dissolution rate. Without oral processing, IDDSI level 3 and 4 lubricants significantly delayed the dissolution of paracetamol tablets. After oral processing, particularly with crushed tablets, there was a substantial increase in the dissolution rate. The in vitro dissolution test is designed for testing whole tablets that arrive in the stomach as an intact unit. It is not ideal for predicting the drug bioavailability of whole or crushed tablets mixed into fluids because it does not account for changes that happen to the fluid’s structure during the oral and swallowing process (i.e., shearing forces, incorporated saliva, body temperature). And so, using an in vitro dissolution test to predict the dissolution rate of medications mixed with thick vehicles, including medication lubricants or thickened fluids is discouraged. Further research is required to explore whether these products significantly affect the rate and extent of drug absorption in vivo.

After oral processing, there were significant alterations in the viscosity and some of the texture features measurements of the medication lubricants with and without crushed paracetamol tablets. A significant decrease in viscosity was obtained, particularly for the thickest product, Gloup Forte, and for the starch-based product (MediSpend), which is susceptible to being hydrolyzed by α-amylase enzyme after coming into contact with human saliva. Therefore, it is necessary to consider ways to incorporate the effect of oral environment and oral and swallowing processing on dysphagia-oriented products, due to their essential influence on the swallowing safety for patients with dysphagia. By understanding the changes that occur to the bolus through oral processing, further insight is gained for developing safer products for managing dysphagia.

## Figures and Tables

**Table 1:** Details of the five medication swallowing lubricants tested in this study. Flavor and composition details are as provided on the product container; IDDSI classification and yield stress are as reported in [14].

Name	Manufacturer	Flavour	Composition	IDDSI Classification	Yield Stress (Pa)
MediSpend	Fagron, Rotterdam, The Netherlands	lemon	Purified water, modified food starch, natural lemon flavour, sodium citrate, citric acid, sucralose, sodium benzoate.	2—mildly thick	2.47
Severo	IMS Medical, Grootebroek, The Netherlands	anise	purified water, cellulose gum, flavour (anise), citric acid, potassium sorbate, aspartame, acesulfame K.	2—mildly thick	-
Gloup Low Sugar	Rushwood, Raamsdonksveer, The Netherlands	raspberry	Water, xylitol, carrageenan, maltodextrin, potassium sorbate, citric acid, colour, aroma.	3—moderately thick	12.50
Gloup Original	Rushwood, Raamsdonksveer, The Netherlands	strawberry/ banana	Water, carrageenan, maltodextrin, potassium sorbate, sucrose, calcium chloride, citric acid, colour, aroma.	3—moderately thick	9.76
Gloup Forte	Rushwood, Raamsdonksveer, The Netherlands	vanilla	Water, dried glucose syrup, sucrose, carrageenan, maltodextrin, potassium sorbate, citric acid, (natural) aroma	4—extremely thick	38.13

- not available.

**Table 2:** The percentage of loss of crushed paracetamol tablets mixed with five medication lubricants with and without oral processing. The data are mean ± se for five replicates.

Loss of Crushed Paracetamol (%)		
Lubricant	without Oral Processing	with Oral Processing
MediSpend	1.20 ± 022	1.78 ± 0.37
Severo	1.47 ± 0.29	1.42 ± 0.21
Gloup Low Sugar	1.88 ± 0.43	2.65 ± 0.55
Gloup Original	1.82 ± 0.98	2.64 ± 0.92
Gloup Forte	4.61 ± 0.97	3.24 ± 0.78
No lubricant (Control)	0.46 ± 0.12	

**Table 3:** The percentage dissolution of paracetamol tablets in simulated gastric fluids at 30 min. Crushed and whole tablets were mixed with five medication lubricants, with and without oral processing, prior to dissolution testing. Mean values were compared with the relevant control (crushed or whole tablets without medication lubricant and oral processing). The data are mean ± se for five replicates.

	Paracetamol Dissolved at 30 Min (%)			
	Crushed		Whole	
	without Oral Processing	with Oral Processing	without Oral Processing	with Oral Processing
Tablets with:				
MediSpend	91.0 ± 0.8	88.6 ± 3.3	88.8 ± 0.3	81.6 ± 3.1
Severo	84.6 ± 4.8	82.6 ± 2.5	85.2 ± 2.3	87.0 ± 3.0
Gloup Low Sugar	63.8 ± 7.4 ***	80.2 ± 2.5	85.8 ± 3.2	83.0 ± 10.8
Gloup Original	49.2 ± 7.9 ***	79.4 ± 5.7	71.0 ± 7.8	81.4 ± 12.8
Gloup Forte	30.6 ± 3.8 ***	42.8 ± 2.3 *	54.0 ± 17.7 *	95.2 ± 7.1
No lubricant (control)	95.8 ± 1.3		91.0 ± 3.1	

*** *p* < 0.001, * *p* < 0.05.

**Table 4:** Texture profile analysis of the medication lubricants with (Y) and without (N) crushed paracetamol tablets, and with and without oral processing. For each medication lubricant analyzed separately using one-way ANOVA with Bonferroni post hoc comparisons, texture characteristic values followed by unlike superscript letters are significantly different (*p* < 0.05). The data are mean ± se for five replicates.

Medication Lubricant	Crush Tablet	Treatment	Texture Characteristic				
			Hardness (g)	Adhesiveness (mJ)	Cohesiveness	Gumminess (g)	Springiness (mm)
MediSpend	N	Control	3.33 ± 0.36 ^a^	0.07 ± 0.04 ^a^	0.85 ± 0.01 ^a^	3.17 ± 0.14 ^b^	19.37 ± 0.22 ^a^
	N	Oral processing	2.26 ± 0.20 ^ab^	0.24 ± 0.18 ^a^	0.47 ± 0.21 ^a^	1.01 ± 0.30 ^a^	15.41 ± 1.56 ^a^
	Y	Control	2.00 ± 0.47 ^b^	0.06 ± 0.01 ^a^	0.44 ± 0.12 ^a^	2.00 ± 0.47 ^a^	11.60 ± 2.00 ^a^
	Y	Oral processing	1.96 ± 0.13 ^b^	0.03 ± 0.01 ^a^	0.55 ± 0.13 ^a^	1.00 ± 0.28 ^a^	16.24 ± 1.63 ^a^
Severo	N	Control	3.33 ± 0.14 ^a^	0.08 ± 0.02 ^a^	0.59 ± 0.13 ^a^	2.00 ± 0.50 ^a^	19.65 ± 0.04 ^a^
	N	Oral processing	1.87 ± 0.36 ^b^	0.02 ± 0.01 ^a^	0.59 ± 0.09 ^a^	2.59 ± 0.33 ^a^	17.66 ± 0.94 ^a^
	Y	Control	1.83 ± 0.14 ^b^	0.02 ± 0.01 ^a^	0.69 ± 0.07 ^a^	1.37 ± 0.15 ^a^	13.97 ± 2.60 ^a^
	Y	Oral processing	1.88 ± 0.15 ^b^	0.05 ± 0.02 ^a^	0.56 ± 0.07 ^a^	1.04 ± 0.20 ^a^	18.86 ± 0.92 ^a^
Gloup Low Sugar	N	Control	4.83 ± 0.68 ^a^	0.12 ± 0.04 ^a^	0.75 ± 0.09 ^a^	3.67 ± 0.73 ^a^	16.79 ± 0.29 ^a^
	N	Oral processing	5.16 ± 0.64 ^a^	0.15 ± 0.04 ^a^	0.72 ± 0.05 ^a^	3.77 ± 0.61 ^a^	18.74 ± 0.44 ^a^
	Y	Control	3.33 ± 0.36 ^a^	0.07 ± 0.03 ^a^	0.80 ± 0.09 ^a^	2.47 ± 0.40 ^a^	12.05 ± 1.48 ^a^
	Y	Oral processing	4.77 ± 0.59 ^a^	0.10 ± 0.02 ^a^	0.78 ± 0.05 ^a^	3.56 ± 0.53 ^a^	18.44 ± 1.52 ^a^
Gloup Original	N	Control	4.33 ± 0.27 ^ab^	0.08 ± 0.02 ^a^	0.93 ± 0.03 ^a^	4.03 ± 0.40 ^a^	19.70 ± 0.06 ^a^
	N	Oral processing	4.43 ± 0.19 ^a^	0.10 ± 0.00 ^a^	0.75 ± 0.01 ^a^	3.26 ± 0.11 ^ab^	16.94 ± 0.52 ^ab^
	Y	Control	2.58 ± 0.60 ^b^	0.09 ± 0.03 ^a^	0.82 ± 0.04 ^a^	2.60 ± 0.39 ^b^	13.13 ± 2.69 ^b^
	Y	Oral processing	4.82 ± 0.22 ^a^	0.10 ± 0.02 ^a^	0.74 ± 0.04 ^a^	3.03 ± 0.32 ^ab^	18.26 ± 0.32 ^a^
Gloup Forte	N	Control	9.17 ± 0.14 ^a^	0.33 ± 0.01 ^a^	0.83 ± 0.01 ^a^	7.60 ± 0.19 ^a^	19.62 ± 0.06 ^a^
	N	Oral processing	9.16 ± 0.25 ^a^	0.26 ± 0.02 ^a^	0.80 ± 0.01 ^a^	7.27 ± 0.22 ^a^	17.32 ± 0.49 ^ab^
	Y	Control	4.50 ± 0.24 ^b^	0.20 ± 0.05 ^a^	0.83 ± 0.04 ^a^	3.87 ± 0.31 ^b^	12.20 ± 2.31 ^b^
	Y	Oral processing	9.37 ± 0.39 ^a^	0.29 ± 0.02 ^a^	0.81 ± 0.01 ^a^	7.27 ± 0.14 ^a^	18.09 ± 0.14 ^a^

## Data Availability

Data will be made available upon request.

## References

[1] Buhmann C., Bihler M., Emich K., Hidding U., Pötter-Nerger M., Gerloff C., Niessen A., Flügel T., Koseki J.-C., Nienstedt J.C. (2019). Pill Swallowing in Parkinson’s Disease: A Prospective Study Based on Flexible Endoscopic Evaluation of Swallowing. Park. Relat. D.

[2] Cicala G., Barbieri M.A., Spina E., De Leon J. (2019). A Comprehensive Review of Swallowing Difficulties and Dysphagia Associated with Antipsychotics in Adults. Expert. Rev. Clin. Pharmacol..

[3] Miles A., McLellan N., Machan R., Vokes D., Hunting A., McFarlane M., Holmes J., Lynn K. (2018). Dysphagia and Laryngeal Pathology in Post-Surgical Cardiothoracic Patients. J. Crit. Care.

[4] Leonard R.J., White C., Mckenzie S., Belafsky P.C. (2014). Effects of Bolus Rheology on Aspiration in Patients with Dysphagia. J. Acad. Nutr. Diet..

[5] Sopade P., Halley P., Cichero J., Ward L. (2007). Rheological Characterisation of Food Thickeners Marketed in Australia in Various Media for the Management of Dysphagia. I: Water and Cordial. J. Food Eng..

[6] Zargaraan A., Rastmanesh R., Fadavi G., Zayeri F., Mohammadifar M.A. (2013). Rheological Aspects of Dysphagia-Oriented Food Products: A Mini Review. Food Sci. Hum. Wellness.

[7] Sestili M., Logrippo S., Cespi M., Bonacucina G., Ferrara L., Busco S., Grappasonni I., Palmieri G.F., Ganzetti R., Blasi P. (2018). Potentially Inappropriate Prescribing of Oral Solid Medications in Elderly Dysphagic Patients. Pharmaceutics.

[8] Drumond N., Stegemann S. (2020). Better Medicines for Older Patients: Considerations between Patient Characteristics and Solid Oral Dosage Form Designs to Improve Swallowing Experience. Pharmaceutics.

[9] Forough A.S., Lau E.T., Steadman K.J., Cichero J.A., Kyle G.J., Santos J.M.S., Nissen L.M. (2018). A Spoonful of Sugar Helps the Medicine Go Down? A Review of Strategies for Making Pills Easier to Swallow. Patient Prefer. Adherence.

[10] Liu F., Ghaffur A., Bains J., Hamdy S. (2016). Acceptability of Oral Solid Medicines in Older Adults with and without Dysphagia: A Nested Pilot Validation Questionnaire Based Observational Study. Int. J. Pharm..

[11] Lau E.T., Steadman K.J., Cichero J.A., Nissen L.M. (2018). Dosage Form Modification and Oral Drug Delivery in Older People. Adv. Drug Deliv. Rev..

[12] Haw C., Stubbs J. (2010). Administration of Medicines in Food and Drink: A Study of Older Inpatients with Severe Mental Illness. Int. Psychogeriatr..

[13] Mc Gillicuddy A., Crean A.M., Sahm L.J. (2016). Older Adults with Difficulty Swallowing Oral Medicines: A Systematic Review of the Literature. Eur. J. Clin. Pharmacol..

[14] Malouh M.A., Cichero J.A., Manrique Y.J., Crino L., Lau E.T.L., Nissen L.M., Steadman K.J. (2020). Are Medication Swallowing Lubricants Suitable for Use in Dysphagia? Consistency, Viscosity, Texture, and Application of the International Dysphagia Diet Standardization Initiative (IDDSI) Framework. Pharmaceutics.

[15] Manrique Y.J., Sparkes A.M., Cichero J.A., Stokes J.R., Nissen L.M., Steadman K.J. (2016). Oral Medication Delivery in Impaired Swallowing: Thickening Liquid Medications for Safe Swallowing Alters Dissolution Characteristics. Drug Dev. Ind. Pharm..

[16] Manrique-Torres Y.J., Lee D.J., Islam F., Nissen L.M., Cichero J.A., Stokes J.R., Steadman K.J. (2014). Crushed Tablets: Does the Administration of Food Vehicles and Thickened Fluids to Aid Medication Swallowing Alter Drug Release?. J. Pharm. Pharm. Sci..

[17] Dressman J.B., Amidon G.L., Reppas C., Shah V.P. (1998). Dissolution Testing as a Prognostic Tool for Oral Drug Absorption: Immediate Release Dosage Forms. Pharm. Res..

[18] Martir J., Flanagan T., Mann J., Fotaki N. (2020). Impact of Food and Drink Administration Vehicles on Paediatric Formulation Performance Part 2: Dissolution of Montelukast Sodium and Mesalazine Formulations. AAPS PharmSciTech.

[19] Ruiz-Picazo A., Colón-Useche S., Gonzalez-Alvarez M., Gonzalez-Alvarez I., Bermejo M., Langguth P. (2020). Effect of Thickener on Disintegration, Dissolution and Permeability of Common Drug Products for Elderly Patients. Eur. J. Pharm. Biopharm..

[20] Onuki Y., Sugiura D., Kumada S., Kobayashi R., Nakamura T., Kogawa T., Sakai H., Okada K. (2022). The Molded Tablet, a Disintegrant-Free Orally Disintegrating Tablet, Resists Thickening Solution-Reduced Drug Dissolution. J. Drug Deliv. Sci. Technol..

[21] Radhakrishnan C. (2016). Oral Medication Dose Form Alteration: Patient Factors and the Effect of Adding Thickened Fluids. Ph.D. Thesis.

[22] Torres Y.J.M. (2015). Understanding the Mechanism of Drug Delivery from Thickened Fluids to Aid Swallowing of Medications. Ph.D. Thesis.

[23] Chen F., Dirven S., Xu W., Bronlund J., Li X., Pullan A. (2012). Review of the Swallowing System and Process for a Biologically Mimicking Swallowing Robot. Mechatronics.

[24] Sharma M., Pico J., Martinez M.M., Duizer L. (2020). The Dynamics of Starch Hydrolysis and Thickness Perception During Oral Processing. Int. Food Res. J..

[25] Ferry A., Hort J., Mitchell J., Lagarrigue S., Pamies B. (2004). Effect of Amylase Activity on Starch Paste Viscosity and Its Implications for Flavor Perception. J. Texture Stud..

[26] Vilardell N., Rofes L., Arreola V., Speyer R., Clavé P. (2016). A Comparative Study between Modified Starch and Xanthan Gum Thickeners in Post-Stroke Oropharyngeal Dysphagia. Dysphagia.

[27] Hanson B., O’leary M.T., Smith C.H. (2012). The Effect of Saliva on the Viscosity of Thickened Drinks. Dysphagia.

[28] Peyron M.-A., Gierczynski I., Hartmann C., Loret C., Dardevet D., Martin N., Woda A. (2011). Role of Physical Bolus Properties as Sensory Inputs in the Trigger of Swallowing. PLoS ONE.

[29] Ryu J.S., Park D., Oh Y., Lee S.T., Kang J.Y. (2016). The Effects of Bolus Volume and Texture on Pharyngeal Pressure Events Using High-Resolution Manometry and Its Comparison with Videofluoroscopic Swallowing Study. J. Neurogastroenterol. Motil..

[30] Chen J. (2009). Food Oral Processing—A Review. Food Hydrocoll..

[31] Stading M., Waqas M.Q., Holmberg F., Wiklund J., Kotze R., Ekberg O. (2019). A Device That Models Human Swallowing. Dysphagia.

[32] Kalantzi L., Reppas C., Dressman J., Amidon G., Junginger H., Midha K., Shah V., Stavchansky S., Barends D.M. (2006). Biowaiver Monographs for Immediate Release Solid Oral Dosage Forms: Acetaminophen (Paracetamol). J. Pharm. Sci..

[33] Bernal V., Erto A., Giraldo L., Moreno-Piraján J.C. (2017). Effect of Solution Ph on the Adsorption of Paracetamol on Chemically Modified Activated Carbons. Molecules.

[34] Nissen L.M., Haywood A., Steadman K.J. (2009). Solid Medication Dosage Form Modification at the Bedside and in the Pharmacy of Queensland Hospitals. J. Pharm. Pract. Res..

[35] Marquis J., Schneider M.-P., Payot V., Cordonier A.-C., Bugnon O., Hersberger K.E., Arnet I. (2013). Swallowing Difficulties with Oral Drugs among Polypharmacy Patients Attending Community Pharmacies. Int. J. Clin. Pharm..

[36] Mandel A.L., des Gachons C.P., Plank K.L., Alarcon S., Breslin P.A.S. (2010). Individual Differences in Amy1 Gene Copy Number, Salivary A-Amylase Levels, and the Perception of Oral Starch. PLoS ONE.

[37] British Pharmacopoeia Commission (2018). Appendix XII Recommendations on Dissolution Testing. The British Pharmacopoeia 2018.

[38] United States Pharmacopoeia (2011). The United States Pharmacopeia and National Formulary USP 34–NF 29.

[39] The International Council for Harmonisation of Technical Requirements for Pharmaceuticals for Human Use (ICH) (1996). Guidance for industry: Validation of Analytical Procedures: Text and Methodology. https://www.ich.org/page/quality-guidelines.

[40] Su M., Zheng G., Chen Y., Xie H., Han W., Yang Q., Sun J., Lv Z., Chen J. (2018). Clinical Applications of IDDSI Framework for Texture Recommendation for Dysphagia Patients. J. Texture Stud..

[41] Hadde E.K., Cichero J.A., Nicholson T.M. (2016). Viscosity of Thickened Fluids That Relate to the Australian National Standards. Int. J. Speech-Lang. Pathol..

[42] Szabó P., Kállai-Szabó B., Kállai-Szabó N., Sebe I., Zelkó R. (2014). Preparation of Hydroxypropyl Cellulose Microfibers by High-Speed Rotary Spinning and Prediction of the Fiber-Forming Properties of Hydroxypropyl Cellulose Gels by Texture Analysis. Cellulose.

[43] Food and Drug Administration (1997). Guidance for industry: Dissolution testing of Immediate Release Solid Oral Dosage Forms. Rockville, MD: US Department of Health and Human Services, FDA, Center for Drug Evaluation and Research. https://www.fda.gov/media/70936/download.

[44] Chen J., Stokes J.R. (2012). Rheology and Tribology: Two Distinctive Regimes of Food Texture Sensation. Food Sci. Technol..

[45] Van Eck A., Stieger M. (2020). Oral Processing Behavior, Sensory Perception and Intake of Composite Foods. Food Sci. Technol..

[46] Janssen A.M., Terpstra M.E., De Wijk R.A., Prinz J.F. (2007). Relations between Rheological Properties, Saliva-Induced Structure Breakdown and Sensory Texture Attributes of Custards. J. Texture Stud..

[47] Dirven S., Xu W., Cheng L.K. (2015). Sinusoidal Peristaltic Waves in Soft Actuator for Mimicry of Esophageal Swallowing. IEEE ASME Trans. Mechatron..

[48] Dirven S., Xu W., Cheng L.K., Allen J. (2015). Biomimetic Investigation of Intrabolus Pressure Signatures by a Peristaltic Swallowing Robot. IEEE T. Instrum. Meas..

[49] Lavoisier A., Shreeram S., Jedwab M., Ramaioli M. (2021). Effect of the Rheological Properties of the Liquid Carrier on the in Vitro Swallowing of Solid Oral Dosage Forms. J. Texture Stud..

[50] Saitoh E., Shibata S., Matsuo K., Baba M., Fujii W., Palmer J.B. (2007). Chewing and Food Consistency: Effects on Bolus Transport and Swallow Initiation. Dysphagia.

[51] Wei Y., Guo Y., Li R., Ma A., Zhang H. (2020). Rheological Characterization of Polysaccharide Thickeners Oriented for Dysphagia Management: Carboxymethylated Curdlan, Konjac Glucomannan and Their Mixtures Compared to Xanthan Gum. Food Hydrocoll..

[52] Himashree P., Sengar A.S., Sunil C. (2022). Food Thickening Agents: Sources, Chemistry, Properties and Applications—A Review. Int. J. Gastron. Food Sci..

[53] Salah R.B., Besbes S., Chaari K., Rhouma A., Attia H., Deroanne C., Blecker C. (2010). Rheological and Physical Properties of Date Juice Palm by-Product (*Phoenix dactylifera* L.) and Commercial Xanthan Gums. J. Texture Stud..

[54] Houjaij N., Dufresne T., Lachance N., Ramaswamy H. (2009). Textural Characterization of Pureed Cakes Prepared for the Therapeutic Treatment of Dysphagic Patients. Int. J. Food Prop..

[55] Steele C.M., Alsanei W.A., Ayanikalath S., Barbon C.E.A., Chen J., Cichero J.A.Y., Coutts K., Dantas R.O., Duivestein J., Giosa L. (2015). The Influence of Food Texture and Liquid Consistency Modification on Swallowing Physiology and Function: A Systematic Review. Dysphagia.

[56] Momosaki R., Abo M., Kobayashi K. (2013). Swallowing Analysis for Semisolid Food Texture in Poststroke Dysphagic Patients. J. Stroke Cerebrovasc. Dis..

[57] Sungsinchai S., Niamnuy C., Wattanapan P., Charoenchaitrakool M., Devahastin S. (2019). Texture Modification Technologies and Their Opportunities for the Production of Dysphagia Foods: A Review. Compr. Rev. Food Sci. Food Saf..

[58] Vallons K.J., Helmens H.J., Oudhuis A. (2015). Effect of Human Saliva on the Consistency of Thickened Drinks for Individuals with Dysphagia. Int. J. Lang Comm. Dis..

[59] Rofes L., Arreola V., Mukherjee R., Swanson J., Clavé P. (2014). The Effects of a Xanthan Gum-Based Thickener on the Swallowing Function of Patients with Dysphagia. Aliment. Pharmacol. Ther..

[60] Vallons K.J., Oudhuis L.A., Helmens H.J., Kistemaker C. (2015). The Effect of Oral Processing on the Viscosity of Thickened Drinks for Patients with Dysphagia. Ann. Rehabil. Med..

[61] Chojin Y., Kato T., Rikihisa M., Omori M., Noguchi S., Akata K., Ogoshi T., Yatera K., Mukae H. (2017). Evaluation of the mann assessment of swallowing ability in elderly patients with pneumonia. Aging Dis..

[62] Maeda K., Akagi J. (2015). Decreased Tongue Pressure Is Associated with Sarcopenia and Sarcopenic Dysphagia in the Elderly. Dysphagia.

[63] Marcott S., Dewan K., Kwan M., Baik F., Lee Y.-J., Sirjani D. (2020). Where Dysphagia Begins: Polypharmacy and Xerostomia. Fed. Pract..

